# Innovative Technologies for Cultural Heritage. Tattoo Sensors and AI: The New Life of Cultural Assets

**DOI:** 10.3390/s20071909

**Published:** 2020-03-30

**Authors:** Maurizio Talamo, Federica Valentini, Andrea Dimitri, Ivo Allegrini

**Affiliations:** 1 INUIT Foundation, Tor Vergata University of Rome, via dell’Archiginnasio snc, 00133 Rome, Italy; maurizio.talamo@uniroma2.it; 2 Sciences and Chemical Technologies Department, Tor Vergata University of Rome, via della Ricerca Scientifica 1, 00133 Rome, Italy; federica.valentini@uniroma2.it; 3 Envint Srl, Via Paradiso 65a, Montopoli di Sabina, 02434 Rieti, Italy; ivo.allegrini@tiscali.it

**Keywords:** cultural heritage, cultural heritage ecosystem, sensors, tattoo sensors, nanoparticles, cellular uptake, RTLS, supervised vs. unsupervised learning, advanced neural networks, causal analysis, smart cities

## Abstract

Conservation and restoration of cultural heritage is something more than a simple process of maintaining the existing. It is an integral part of the improvement of the cultural asset. The social context around the restoration shapes the specific actions. Today, preservation, restoration, enhancement of cultural heritage are increasingly a multidisciplinary science, meeting point of researchers coming from heterogeneous study areas. Data scientists and Information technology (IT) specialists are increasingly important. In this context, networks of a new generation of smart sensors integrated with data mining and artificial intelligence play a crucial role and aim to become the new skin of cultural assets.

## 1. Introduction

“Cultural heritage is the legacy of physical artifacts and intangible attributes of a group or society that are inherited from past generations, maintained in the present and bestowed for the benefit of future generations” (www.unesco.org).

Cultural heritage includes tangible culture (such as buildings, monuments, landscapes, books, works of art, and artifacts), intangible culture (such as folklore, traditions, language, and knowledge), and natural heritage (including culturally significant landscapes, and biodiversity).

In this context, it is impossible not to remember or deny the role of the absolute leader of the Italian cultural heritage. Italy is considered the birthplace of Western civilization and a cultural superpower (Arab news, The Global Times, The Australian, etc.). Italy is home to the greatest number of UNESCO World Heritage Sites (54) to date, and according to one estimate, the country is home to half the world’s great art treasures. Overall, the nation has an estimated 100,000 monuments of any sort (churches, cathedrals, archaeological sites, houses and statues).

Conservation and restoration of cultural heritage is something more than a simple process of maintaining the existing. It is an integral part of the improvement of the cultural asset. The social context around the restoration shapes the specific actions.

“This intangible cultural heritage, transmitted from generation to generation, is constantly recreated by communities and groups in response to their environment, their interaction with nature and their history, and provides them with a sense of identity and continuity, thus promoting respect for cultural diversity and human creativity.” (UNESCO 2003)

Cultural heritage is one of the main attractors of tourism from all over the world. It strongly influences the economy of a city and its management policies. Sustainability of conservation and restoration policies cannot ignore but have to strongly consider all recalled aspects. They define requirements of the conservation and restoration policies: these policies have to be deeply “personalized” to the asset, non-invasive (to avoid closures of places and areas) and, for the same reasons, strongly prevention-oriented.

Today, preservation, restoration, enhancement of cultural heritage are increasingly a multidisciplinary science, meeting point of researchers coming from heterogeneous study areas. Data scientists and Information technology (IT) specialists are increasingly important.

In this context, networks of smart sensors integrated with data mining and artificial intelligence, play a crucial role. In this paper, we identify a data-driven approach based on direct and continuous sensor data to assess the impact of the surrounding environment and physiological changes.

Recently, Smart sensors are continuously required in the cultural heritage field applications (diagnosis and restoration/preservation steps), especially for “in situ” monitoring (when in presence of immovable cultural heritage objects and surfaces), for non-invasive and no-destructive treatments (applied towards historical surfaces) and finally, for a quick data analysis, thanks to the Wireless transduction of the signals [1]. The advancement in sensor device technologies implies novel miniaturized tools (i.e., electrochemical and electronic portable instruments); innovative highly sensitive layers [2,3], selective and specific for the molecular recognition of artwork target probes and also high resolution algorithms, capable of analyzing big data (especially coming from the continuous monitoring campaigns, which transmit data from remote control systems).

Furthermore, the great opportunity to assemble hyphenated techniques [4], as NMR-MOUSE [5], neutrons [6] physical approaches represents a future challenge in cultural heritage restoration and conservation/valorization management. Only this multi-disciplinary approach could achieve impact and success for a global characterization of artwork objects and valorization of archaeological surfaces, providing very useful information to restorers and conservator scientists when they approach in terms of experimental restoration and conservation treatments.

The Internet of Things (IoT) paradigm together with a new model of artificial intelligence allows us to capture the right dimension of the cultural heritage: sensors intercept a living body defined by the cultural asset and its environment. Sent data, opportunely elaborated, allow mining its sensations. This means that we need a causal engine able to keep and analyze together physical data (gas measurements) with social data coming from the context where the cultural asset is located.

We describe a model to investigating the potential of fusing on-body physiological signals, environmental sensory data and historical data in order to achieve the following objectives: (1) to shape the long term impact of the ambient environment on a cultural asset, (2) borrowing the ‘precise medicine’ approach, to define actions to avoid damages and to minimize the spatial range of an intervention [7].

In Section 2, the model for precise anomaly detection and risk management has been introduced. Three main properties characterize the model are:

1. a new family of smart portable sensors tattoo, designed here for the analysis of cultural heritage surfaces. These tattoo prototypes present two innovation aspects, as the miniaturize tools, due to the high technological printers, and highly sensitive nanostructured layers, for a selective molecular recognition pathways on the tattoo sensors transducer.
2. the capability of the monitor system to become the new skin of the cultural assets and then their interface with the environment; the full integration with the ecosystem that the cultural heritage defines in an area. The goal is ambitious also in another sense: the monitoring system is more than transparent: it enters in the ecosystem proving it with new opportunities. This is possible thanks to a causal engine able to integrate heterogeneous data (physical and social data);
3. a complex and complete model for monitoring a cultural asset, considering all aspects behind a distributed monitoring system: a distributed network of sensors, communication channels, data and data analysis tools. The proposed model combines all properties, together.

In the following sections, we enter in the details of each component of the model. In Section 3 a new family of smart portable sensors tattoo, designed and proposed for the analysis of cultural heritage surfaces is presented. In Section 4, the communication layer, the network that allows all components to interact to output relevant events and causalities have been analyzed, in detail. In Section 5, the causal engine and the risk prediction system have been proposed and explained. In Section 6, two experiments (the first outdoor and the second in a gallery) to better understand the potentialities of the causal engine, have been presented and in the last paragraph, the general conclusions have been summarized.

## 2. A Model for Precise Anomaly Detection and Risk Management: How to Keep All Together

**Figure 1:**
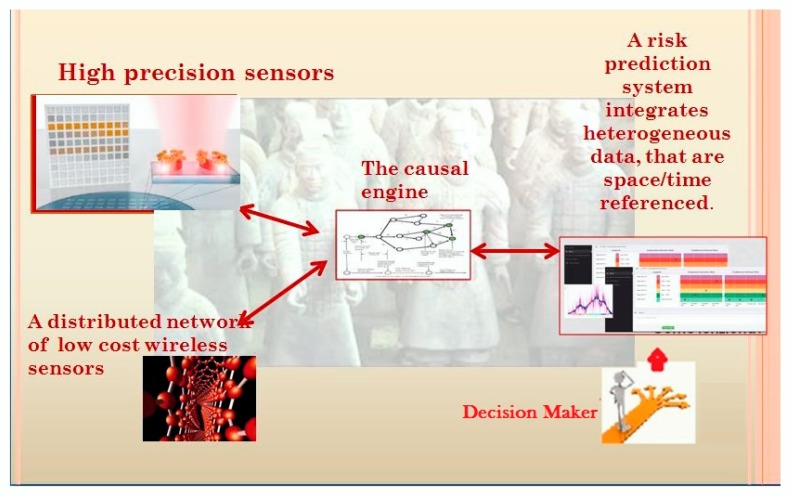
The proposed monitoring architecture. Data coming from an integrated system of few high precision sensors and many low-cost simple sensors will be sent to the causal engine able to discover causal relationships. The last component is the risk prediction system, with a graphical interface. It allows querying the monitored area to understand evolutions of a prefix (an ordered sequence of events) and to help the decision maker.

Given a target area where is located a system of cultural assets we developed and propose a complete monitoring architecture that assembles all components showed in Figure 1:

1. A small network of high precision and high performing sensors allocated in the target area. Small means a network of few nodes.
2. A distributed network of low-cost wireless sensors.
3. A causal engine, based on a newly-tested mathematical model of artificial intelligence that receive data from all sensors, the system integrates heterogeneous data, which are space/time referenced, and process them capturing relationships.
4. A Risk Prediction System (RPS). It starts from a prefix, i.e., a sequence of events spatially and temporally defined, and querying the causal engine, defines a set of future scenarios, evolution of the starting prefix. The RPS has a graphical interface, able to collect user-defined prefixes, or able to capture from a context the current prefix. The same user interface shows evolutions of the prefix and allows us to understand risks and to lead decisions.

High precision and high performing sensors allow reproducing with high precision the profile and temporal behavior of the area where they are located.

Collected data train an expert system with the role to recognize and classify the events coming from all low-cost sensors distributed around the urban area.

High precision and high performing sensors capture the concept of aggregated evolution of an ecosystem, where many causal agents interact determining what we know and see as the “normal” behavior of the ecosystem.

**Figure 2:**
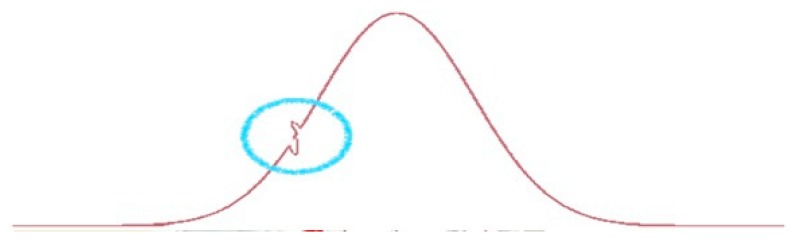
The integration of data coming from the set of sensors (high precision and high performing sensors and local sensors) allows calculating the expected value in a location and in a specified time. A spike in the data coming from the local sensor defines an anomaly behavior that could require a decision.

Local sensors capture spikes in the global evolution and allow capturing rare events and associated reversal trends (Figure 2).

As a result of this interaction, the dataset appears homogeneous and represents a population of space-time trajectories that is the input for the causal analysis.

**Figure 3:**
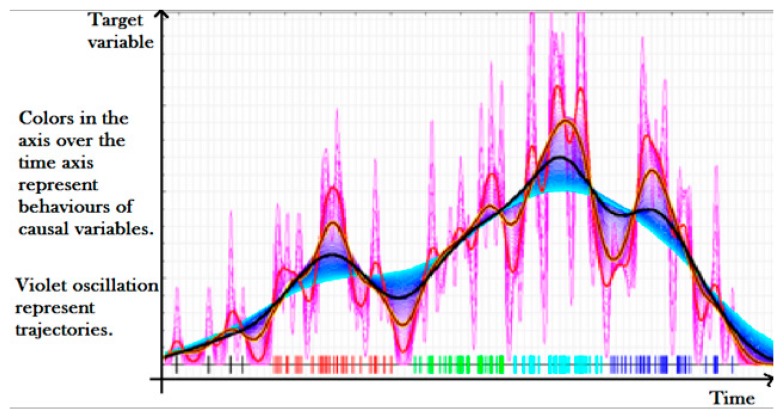
The trend behind the normal evolution represented by a sequence of events can be the result of many elementary events, uncorrelated and with opposite effects. The causal engine is able to isolate them and then to allow a causal analysis and to detect causal evidence behind an event or a behavior.

A probabilistic model, starting from the rules of a random biological system, step by step, moves on a “target” biological system and predicts its behaviors and trajectories in the space and in the time directions (Figure 3).

Two aspects have to be underlined: first, the proposed architecture allows us to preview an evolution of a variable but also to understand causal relationships behind that evolution and, then, to guide actions to preserve the cultural asset. Secondly, the capability to forecast the evolution of a trajectory allows managing discrete, nonlinear deterioration variables and, from another point of view, gives a precise definition of the rare event and how to manage it. To be clearer, let us consider an example: a rare event is a fire. Let us consider an asset that usually, in a healthy state, resists a weak fire. A slow form of pollution changes this resistance. Until a fire occurs, there are no visible effects of the pollution. The fire event will damage the asset wherever in the previous fire event nothing happened. Traditional statistical models fail to manage these rare events [8]. Our model fills these gaps [9,10].

The model has general-purpose architecture and applications have been done in the field of the protection and enhancement of the cultural heritage [11] but also in other knowledge fields [10,12]. the third parts used the causal engine to understand causality in their experiments [7,13].

In the following, we analyze the details of each one of the components of the introduced architecture.

## 3. Sensors Allow Tasting Sensations in A Living BODY

We said that the IoT paradigm allows us to capture the right dimension of the cultural heritage: sensors are a key element in this direction. The correct perspective is to see them as interceptors of a living body (the cultural asset) and captured and sent data have to be viewed as the sensations of this body. The challenge is to develop light sensors, virtually transparent, and able to capture all the internal and environmental parameters of the object of monitoring.

According to this, it is important to consider a new generation of sensors, highly performing to be applied in a wide range of fields: from medicine to cultural heritage. Recently, advanced technologies provide miniaturized smart devices suitable for nanomedicine [14], as nano implantable sensors, nanomotors, nanomachines and nano-antennas suitable for the wireless transmitting signals, generated by the monitored biological systems. Actually, the ‘tattoo sensors’ seem to be very promising for nanomedicine, considering their great connectivity with the epidermal tissues, where they can act not only as a sensorial tool, but also as an actuator devices. Especially, these latter are suitable for the controlled releasing of pharmacological agents such as active principles (contained in drugs) for carrying out targeted therapies, towards specific organs and cellular compartments. The sensor tattoo philosophy has developed a great deal by welcoming the technological innovations in the field of electronics, electrochemistry, screen printing technique up to manufacturing versatile devices also for the environment and for the cultural heritage contained therein [15].

The exciting compatibility of new sensors tattoo prototypes, exhibited towards the historical and archaeological surfaces and artwork objects, depends on the innovative nanomaterials, specially made and designed for cultural heritage surfaces. Among these new nanostructured materials, there are carbon nanostructures (i.e., graphene), metallic nanoparticles, nanostructured biopolymers and nanocomposite materials [11]. These latter offer many advantages, mainly the “same chemical composition” of the artwork objects, which need to be repaired and preserved, but with the advantages of the nanoscale dimensions [11]. Among the nanoscale size advantages, the solubility of the innovative nanomaterials concerns unusual working medium/solvents, which however are extremely compatible with the surfaces of cultural heritage. A further property is an optical feature since these nanomaterials do not alter the color of the historical–artistic surfaces, after the restoration and conservation treatments. Both of the cited properties/features, reported above, mainly depend on the quantum confinement effects of matter, at the nanoscale levels.

The great opportunity to combine smart nanomaterials [16,17], especially having a higher surface nominal area [18,19] with advanced and modern Nanotechnologies, provides new movable tools suitable for diagnosis and conservation of artwork objects, directly in situ working. Mainly, the new screen printing techniques [20,21], i.e., the 3D printing of objects, can be able to fabricate miniaturized devices also equipped with wireless and/or Wi-Fi connections/transmissions of the analytical signals, coming from the surfaces of historical and artistic places. The nanomaterials/nanotechnologies assembly provides two important performances in cultural heritage fields, as in the local diagnosis of artwork material damages and, at the same time, the online monitoring of the environmental agents, responsible for the chemical-physical and biological deterioration events (that occur on historical surfaces, mainly provoking dark patinas and black crusts).

**Figure 4:**
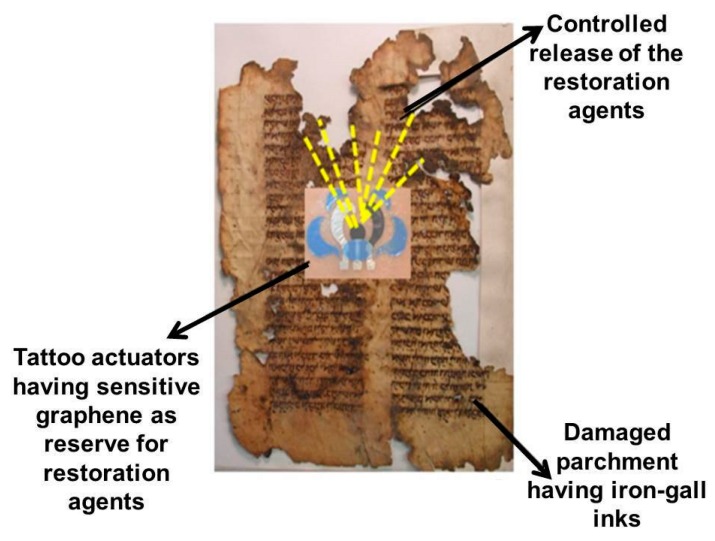
This cartoon (assembled by the authors of the manuscript) represents the main idea of the tattoo actuator mechanism on CH sample, based on functionalized graphene nanomaterial, which acts as a reserve of restoration/consolidation chemical agents, suitable to release the selective agents in a controlled way, on damaged artwork surfaces. These latter samples (i.e., damaged paper and parchment samples) provide the right clock for the modulated releasing of restoration agents, depending on the ΔpH, Δμ and other modification of chemical–physical parameters, induced by inner and/or outer deterioration events.

Furthermore, the possibility of loading tattoo sensors with reserve chemical reagents, allows the device to become an intelligent actuator (see Figure 4), capable of releasing the cleaning, restoration and / or consolidation agent, in a controlled manner/way.

**Figure 5:**
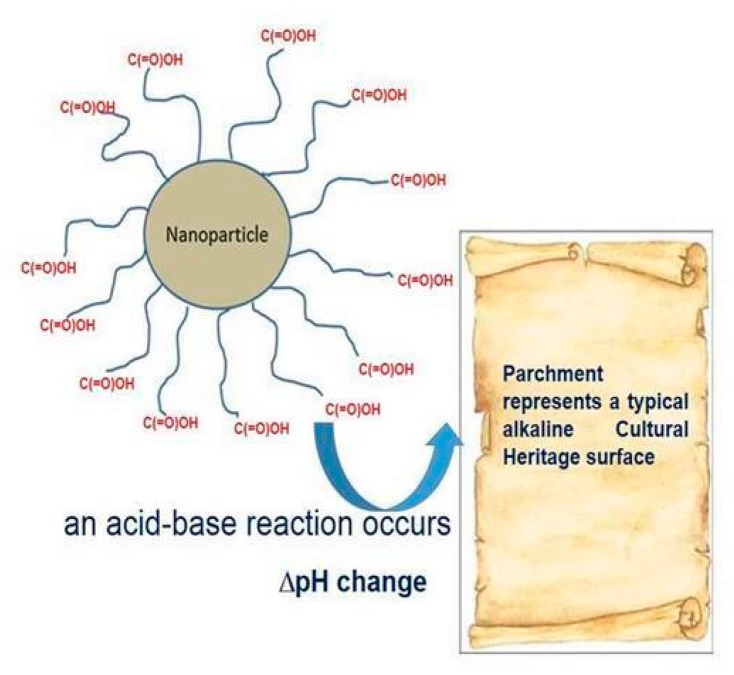
A scheme of the organic functional groups, present on the nanoparticle surfaces, applied for the tattoo sensor assembly and their acid-alkaline based reaction mechanism, suitable to explain the actuator’s main role.

**Figure 6:**
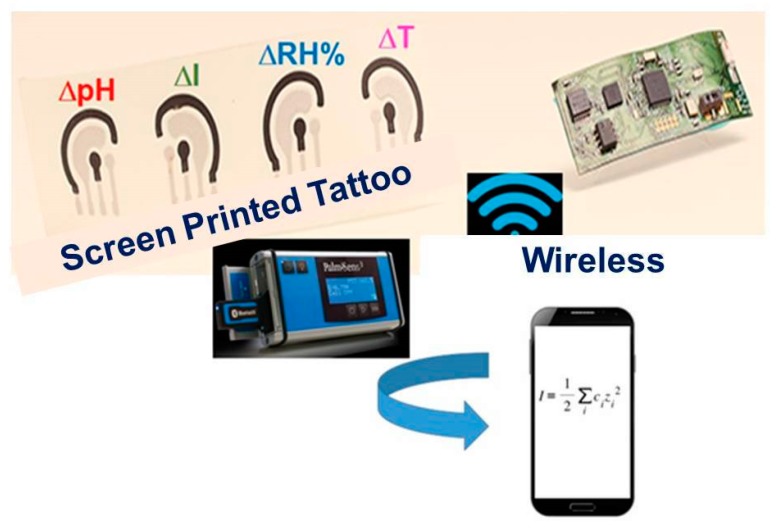
A scheme of integrated tattoo sensors electronic array, directly put in contact with the cultural heritage surfaces. This array is also equipped with a movable Palm–Sens Potentiostat instrument for a wireless recording of the analytical signals, generated by a change of several parameters, as ΔpH; ΔI; ΔRH (%) and ΔT, respectively.

These new prototypes can be assembled with highly selective and specific nanomaterials, ranging from graphene, carbon nanohorns/nanoparticles, metallic nanowires and/or nanocomposite polymeric materials [22], extremely performing thanks to the functionalization of their surfaces [23]. These innovative actuators in cultural heritage field applications are able to release the active restoration/consolidation agents, especially when a change in pH, ionic strength (I = $\frac{1}{2}\;\sum_{i=1}^{N}C_{i}\;Z_{i}{}^{2}$), relative humidity, temperature, optical density in the surrounding environment and/or on the surface of the cultural heritage itself, can occur. The release is controlled precisely by the presence of functional groups on the surfaces (Figure 5) of the nanomaterials, which are extremely sensitive to the variation of the cited chemical-physical parameters [24]. Furthermore, these tattoo sensors are integrated into microcircuits equipped with electronic systems capable of activating the wireless transfer of the analytical signal (Figure 6). In this way, the actuator is also a sensor and it is possible to monitor the restoration and consolidation action over time and space, thanks to the mapping generated by the tattoo sensor system.

**Figure 7:**
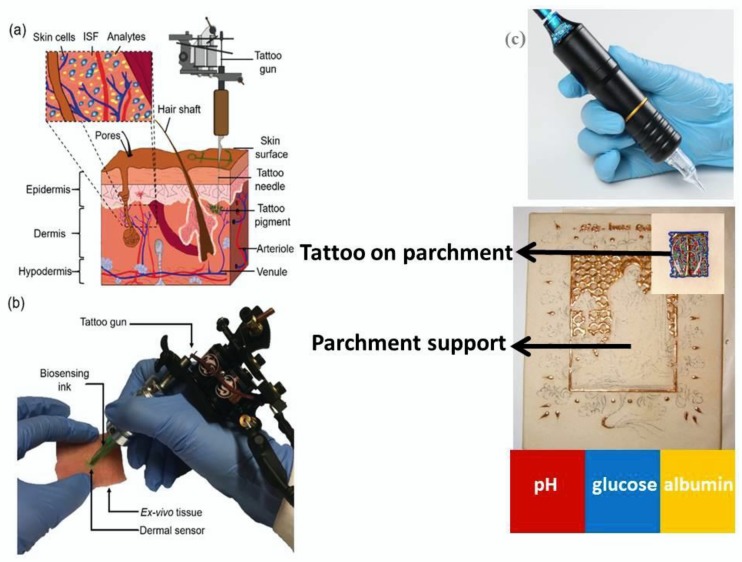
Injection of colorimetric biosensors within the dermis. (**a**) Schematic of human skin cross-section. (**b**) Injection of biosensors in ex vivo porcine skin tissue, reproduced by the permission of [25]. (**c**) A scheme of possible tattoo sensor assembly on parchment, and/or paper support, suitable for the monitoring of several chemical parameters (very useful for the evaluation of the conservation status of ancient manuscript heritage), such as pH, glucose and albumin molecules.

Other authors, [25] in literature, develop innovative and smart tattoo devices, injecting several inks and/or dye pigments into the dermis (Figure 7a,b reproduced and reprinted by the permission of the authors in reference [25]). The resulting tattoo based biosensors are applied for the monitoring of biochemical metabolites, as glucose, albumin and variation of pH parameter values [25]. Originally, this tattoo prototype has been assembled to detect the acid–base homeostasis, diabetes, and liver failure in point-of-care settings.

In the indoor cultural heritage field applications (as archives, museums, libraries), these devices could be also applied directly on the ancient paper manuscript surfaces, parchments and leather coats, in order to monitor (in real-time and in wireless mode/approach) the pH change due to the presence of iron-gall inks, or also induced by the anomalous presence of atmospheric gaseous acidic pollutants (as HNO_3_, HNO_2_, H_2_SO_4_, HCl, etc.) Figure 7c.

Furthermore, this biosensors tattoo could be also applied for the monitoring of the glucose concentration profiles, on ancient historical paper manuscripts, especially when these latter undergo the phenomenon of depolymerization due to natural aging, and/or the anthropogenic pollution, and/or the oxidation processes of cellulose chains and also the acidic hydrolysis of the glucose monomer, assembled into the cellulose chains.

Finally, another interesting biochemical parameter, essential to establish the conservation status of ancient books and manuscripts and also to identify/to trace back the execution technique of the artwork objects, is also the albumin target/probe. This latter was widely used, historically, as a binder (necessary to prepare at the moment or keep it constantly moist) also together with the animal glues (parchment glue, leather glue, fish glue and bones) and egg yolk.

Another interesting nanostructured based tattoo tools, in cultural heritage field, is the combination with the Nuclear Magnetic Resonance (NMR) apparatus, recently applied in cultural heritage [26] for the molecular recognition of chemical species involved in the colored pigment compositions, inks, protective films, dyes, etc. The same authors [26] demonstrate that the chemical-physical interactions between the organic pigments and graphene nanosheets (widely applied for the tattoo assembly) provoke significant changes in spin–spin coupling, the vast increase in relaxation, line broadening and decrease in NMR peak heights. The nature of these changes in NMR profiles of organic dyes and pigments strictly depends on the kind of interactions with graphene and graphene derivatives toward the organic compounds (present in the original materials of cultural heritage objects and surfaces).

Binding to graphene was selective for positively-charged organic assemblies, weaker for non-aromatic and negligible for strongly-negatively-charged molecules, presumably repelled by a negative zeta potential of graphene in water.

**Figure 8:**
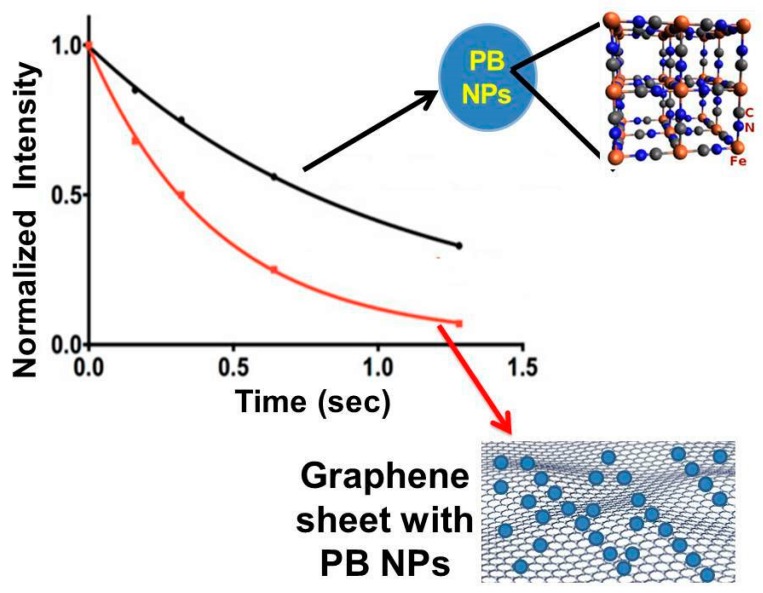
This is a typical scheme of how the relaxation times of organic molecules, such as colored pigments, vary as a result of interaction with graphene. For example, Prussian Blue, which represents a very well-known pigment, shows much longer relaxation times, when it is conjugated with graphene, as the relaxation process is slowed down by interaction with graphene nanosheets. Red and black line profiles in the graph refer to the Prussian Blue nanoparticles conjugated with a graphene nanosheet (red line), and Prussian Blue nanoparticles with its specific lattice, without conjugation with graphene sheet (represented by the black line), respectively.

The fundamental nature of these different electronic interactions between organic and polyaromatic carbon with graphene and graphene derivatives is considered with relevance to the final NMR spectrum profiles, where:

1. in the presence of strong interactions, mainly due to the higher surface area exhibited by graphene toward the adsorbed molecules, relaxation times are rather slow (Figure 8);
2. in the presence of weak interactions, relaxation times are rather rapid (Figure 8);
3. in absence of interactions, no significant modifications are obtained on the spectrum and the NMR profile is the same as that of the original molecules (not interacting with graphene).

**Figure 9:**
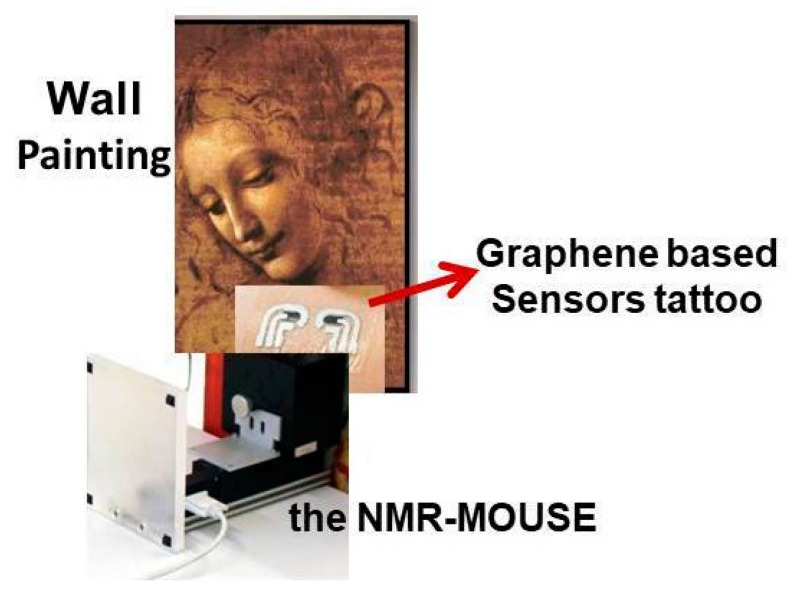
A scheme of the most realistic assembly of the portable Nuclear Magnetic Resonance (NMR) apparatus and the graphene-based working sensor tattoo, put directly in contact with the wall painting areas, for in situ measurements. The main role of the graphene sensor tattoo is to pre-concentrate/to extract the pigments, organic dyes and inks, essential for a high-resolution acquisition of the NMR spectral profiles, having a high signal- to-noise ratio and a high sensitivity. The NMR-MOUSE images have been reproduced and reprinted with the permission of reference [27].

In Figure 9, a more likely representation of the measuring movable/portable apparatus, directly in contact with the cultural heritage surfaces, involving the combination of tattoo sensors and the small portable NMR instrumentation (i.e., the NMR-MOUSE, [27]), has been also highlighted. Here, the key role is played by the tattoo sensor which, by concentrating on its sensitive graphene layer, the pigment and/or ink and/or dye molecules it facilitates the interactions among the organic molecules and the graphene platform. In these conditions, the NMR probe could be directly in contact with a tattoo-based graphene surface enriched with the pigment molecule, and/or with dye, and this enrichment entails a greater analytical sensitivity in the recording of the output NMR signals, and also a better signal to noise ratio.

Other possible combinations and applications of the graphene-based sensors tattoo with the portable NMR apparatuses (commercially available) could concern the analytical diagnosis of damaged stones, plasters and other historical/archaeological areas. In this context, two main research activities could be designed, as:

1. sensors tattoo/movable NMR for diagnosis of the environmental dark crust/patinas, deposited on artwork surfaces (as gaseous pollutants and particulate matter, come from the troposphere);
2. sensors tattoo/movable NMR for analytical evaluation of the cleaning/restoration/conservation/consolidation based treatments of outdoor cultural heritage objects and surfaces.

**Figure 10:**
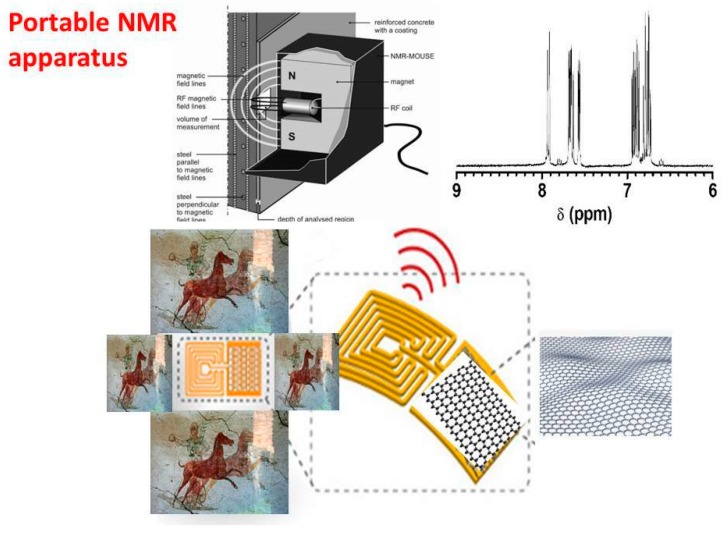
Graphene-based tattoo/combined with the movable NMR apparatus, for several applications: sensors directly applied on cultural heritage surfaces (to identify the composition of black crust layers); actuators suitable to remove environmental black crust/patinas and to provide (for example, as a case study) free water molecules, having short relaxation time, essential to produce a significant NMR signal profile/spectra.

Especially in the first case study, when environmental dark patinas are deposited on historical surfaces with a consistent thickness that contributes to damage these art-work surfaces, the NMR quantitative parameters as the longitudinal relaxation time (T_1_), the effective transverse relaxation time (T_2eff_), and the self-diffusion coefficient (D) of the nucleus, significantly change during the NMR experiments and measurements [26,27]. For example, in the case of water molecules, T_2_ resulted shorter than T_1_, meaning that water molecules, entrapped into dark crust/patinas reduced their motion for the presence of the network due to the deposited layers of environmental dark patinas. In this case, the main role of graphene-based tattoo devices could be related to the adsorption and removal mechanisms, provided by the graphene nanoplatform/sink toward the black crust layers. This is reliable considering that the hydrophobicity of pristine graphene sheets (without polar and ionic chemical functional groups) induce the π–π interactions [28] with the organic chemical compositions of dark crusts, capturing them into the graphene sheets (see Figure 10). In this way, graphene-based sensors tattoo could act as:

1. smart adsorbent carbon-based nanostructured cartridges for removal of black crust (from sensors toward actuators for cleaning of the historical surfaces/stones);
2. smart highly carbon-based electrochemical sensors tattoo for the qualitative and quantitative analysis of the chemical composition of dark crusts, equipped with a wireless transduction of the output analytical signals;
3. smart tattoo actuators able to provide in situ NMR experiments/measurements, before and after the dark patinas removal/remediation (this latter carried out by the adsorption mechanism induced by the hydrophobic graphene sheets).

**Figure 11:**
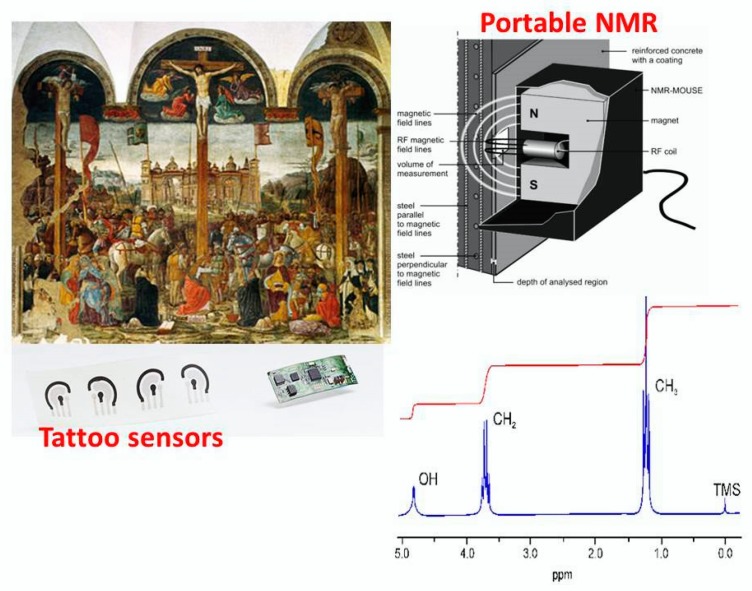
A combination of tattoo sensors and the NMR portable apparatus for the removal of gels and polymers, applied as cleaning agents on wall paintings, provides tattoo actuators, able to absorb the residual cleaning agent (i.e., gels and polymers), providing shorter relaxation times, essential to successfully acquire very well resolved NMR spectral profiles. The Leonardo da Vinci Portrait of the Dukes of Milan with their sons: mixed technique, 90 cm (base) each Convent of Santa Maria delle Grazie, Milan. Permission details: this is a faithful photographic reproduction of a two-dimensional, public domain work of art. The work of art itself is in the public domain for the following reason: his work is in the public domain in its country of origin and other countries and areas where the copyright term is the author’s life plus 100 years or fewer. The official position taken by the Wikimedia Foundation is that “faithful reproductions of two-dimensional public domain works of art are public domain”. http//www.En.wikipwdia.org/ink/The_Last_Supper_(Leonardo)# for the Copyright license permission.

Furthermore, the absorbent action of the graphene actuators also makes it possible to recover the paramagnetic impurities, naturally contemplated in the mineralogical and chemical composition of stones, clays and other rock materials (of historical-artistic interest and importance). This allows the successful NMR experiment to be carried out, thanks to the recovery of short longitudinal relaxation time (T_1_) and the effective transverse relaxation time (T_2eff_), necessary to acquire coherent and statistically significant NMR spectra and profiles (toward chemical species interesting for the historical surface characterization, by applying NMR instrumentation). Another application of graphene-based sensors tattoo and NMR apparatuses (combined and assembled together) consists on the evaluation efficiency of the cleaning and restoration procedures, applied on cultural heritage surfaces. Especially, during the last years, several gels (hydrogels, polymeric gels, etc.) have been developed, characterized and applied on artwork surfaces and objects in order to clean and repair/preserve these historical surfaces. The mechanical action of gels is quite similar to that exhibited by the environmental dark patinas, toward water molecules entrapment (into the gel network systems). The entrapment of the water molecules causes a slowdown in the motions of the nuclei due to the interaction of the water molecules with the surrounding gel/polymers network. This results in a significant elongation of the longitudinal relaxation time (T_1_) and the effective transverse relaxation time (T_2eff_), providing an NMR spectrum and a signal profile without adequate analytical resolution of the outgoing signals. Finally, the great opportunity to combine graphene-based tattoo sensors with the NMR apparatus allows to absorb the gel fractions not removed after the cleaning of the historical surfaces and, at the same time, makes water available (and also other intrinsic molecules, also constitutive of the historical supports, under investigation) to characterize the historical surfaces, before and after cleaning and restoration strategies (Figure 11).

This approach, consisting on the assembly and combination of smart nanomaterials sensors tattoo and portable NMR apparatuses, could be extremely useful in presence of un-movable and un-tangible artwork objectives and surfaces, avoiding and minimizing all the problems related to the CH transport, securing of the artwork objectives, especially when these CHs are removed from places, mainly dedicated to their conservation [29].

## 4. The Communication Layer

These movable sensors allow smart wireless transmission of the signals. This last aspect could be very promising for end-users (as restorers and conservator scientists), especially when there are continuous monitoring campaigns that can take mostly long term experiments, up to several months of continuous measurements.

One of the most important questions in conservation and restoration of artistic sites is the need to collect and transport a large amount of data using low-cost systems to locate in big areas. Sometimes these areas are outdoor areas without pre-existing electricity and communication networks.

Recently, the Information and Communication Technology (ICT) systems seem to be the best solution for a remote programming of the operation of portable sensors, as well as for the management of big data, collected for long times environmental monitoring of CHs. ICT also uses a sophisticated Internet of Things (IoT) system, extremely advanced, with which it allows end-users to manage the operation of sensors and the collection of the corresponding data, remotely and directly from a handheld interface, similar to that exhibited by a Smartphone device.

**Figure 12:**
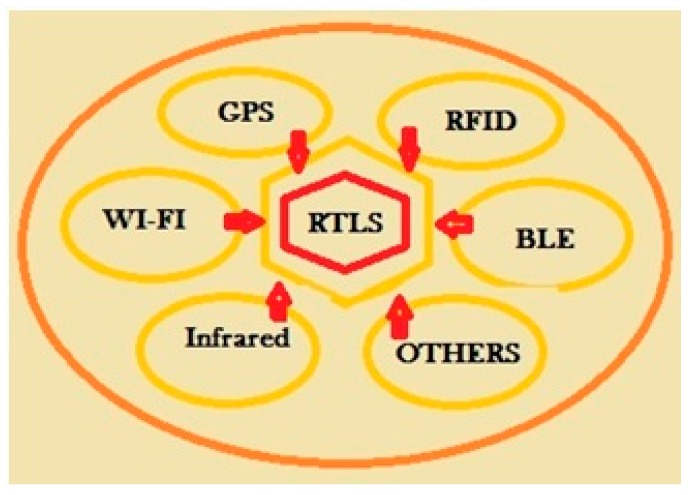
Current real-time location systems are based on wireless technologies, such as Wi-Fi, Bluetooth, ultrawideband, radio frequency identification (RFID), and GPS.

Basic technologies are based on Wireless Sensor Networks (WSN), Bluetooth Low Energy (BLE), radio frequency identification (RFID) and Near Field Communication (NFC). All these technologies seem to be useful; none of them can replace all the others (see Figure 12). For this reason, Real-Time Locating Systems (RTFS) allow a complex mix of previous recalled technologies [30].

The rapid evolution of internet of things requires its application in many heterogeneous environments and it is not ready a communication technology able to manage every one of them. For these reasons, real-time location systems have to include a well-composed mix of communication technologies (e.g., Wi-Fi, wireless sensor networks, active and passive RFID, NFC etc.) with a rich middle-level configuration language and different localization methods and hardware devices associated (e.g., range free vs. range-based, time-difference based, proximity-based and so on). In many application requirements, satisfaction needs not only the capability to configure each technology differently in terms of hardware and software parameters, used protocols and so on, but also parameters that consider interaction with other devices for the same services. What has to be considered is the importance of these aspects. Communication problems, in a network that moves commands more than information, can result either in catastrophic consequences for system safety or in a significant loss of money. New scenarios make the design of an RTLS and, more generally, of a cyber–physical system, a critical challenge [30,31].

## 5. Artificial Intelligence and Causal Analysis

Last but not least, artificial intelligence algorithms and causal analysis have to provide results in real-time regarding the beginning of a deterioration process and locate causes of that process. There are aspects where data mining techniques need to be specialized to CH. The uniqueness and irreversibility of the cultural asset require a fast understanding of deterioration. Identifying the cause allows to extend a measure to all the objects with the same effect. We have to capture a rare event that causes a trend reversal with a hidden absolute effect (the anomaly).

Many of the economic, social and environmental challenges that technological progress is facing have a strong effect on the architecture of a cultural area. Rapid tourist population growth, number of schools, shrinking and growing cities, climate change, biodiversity loss, and other social factors are some of the major issues affecting cultural areas in the urban environment. In this complex network, the objective of making these locations sustainable is, first of all, an understanding challenge of where many factors interact to produce complex chains of events and associated effects. Classical planning tools, in this scenario, become quickly obsolete for an understanding of the effects of the interaction between single groups of actions associated with single policies.

In this scenario artificial intelligence is the right tool with three goals in mind:

1. understanding all effects of an intervention, in a planning phase;
2. understanding effective causes behind a perceived or/and measured effect, for example in terms of pollution, or in terms of mobility traffic and so on;
3. understanding the temporal evolution of a complex set of actions and their interaction and its effect for a specified variable (the presence of a certain substance in the air in a fixed hour of the day; street traffic, noise sound effect, and so on).

To effectively work, an intelligent engine needs data. We develop an intelligent causal machine, Race [10,12] integrated into an efficient sensors network to collect data and process them in real-time.

In a new mathematical framework, based on Kolmogorov complexity, we manage this problem of deep learning. The output is a physical model based on a complex interaction between few high precision sensors and a related set of networks of low costs simple sensors. This allows building a risk manager with a higher level of sharpness and precision that becomes an effective tool for the decision-maker (see next paragraph).

The mathematical primitives behind this engine have been developed considering a specific domain: a predefined set of variables. The main goal of the learning machine is to build a multi-domain risk prediction system allowing changing the set of analyzed variables, considering the available data. The approach to the data analysis has been based on Kolmogorov complexity theory [32] that appeared to be a promising approach in the starting phase of the research [9]. In short, all available data for a cultural asset are considered as an incomplete description of the “life” of it from which—if complete, and with a full understanding of causal relations—the individual risks for the asset could be predicted essentially deterministically. The available data, of course, will not be able to provide a complete picture for the asset as relevant information must be assumed to be missing and causal relations must be discovered [33]. In contrast to purely statistical analysis the approach followed, however, is able to explicitly accommodate this unavoidable lack of knowledge.

For each individual, a set of virtual cultural assets sharing certain key data (including historical data and construction material data of the asset) is modeled: each virtual asset may evolve in different directions from the other ones. We call “trajectory” a path that leads through key events: each trajectory represents the history and the future of a virtual asset. This model then calculates the degree of similarity (in terms of homogeneity, entropy, etc.) of the trajectories for the paths before and after the last key event for the actual asset. Three model parameters have been evaluated in this context: the homogeneity of the trajectories before each key event (confidence), the homogeneity of the trajectories after each key event (causality) and the probability of other risk events in the future. The model enabled us to explore different causal hypotheses for example based on the building material of the cultural asset and clinical data that alter and modify the trajectories between the key events and the values of the model parameters. Overall, the best causal hypotheses should increase the homogeneity of the trajectories for most assets at the end of the study leading to better predictions of future outcomes and necessary follow-ups by the expert (for example the archaeologist).

The model, presented here, goes beyond the state of the art for classical non-Bayesian models since these can only analyze casual relationships for very few parameters and not for the complex causal graphs that underlie the asset trajectories discussed here [33].

It is important to note that with this approach it is possible to retain the entire assets population in the analysis, with expected outputs in the field of meta-analysis. This means that a monitoring analysis in an area could be easily reused in another area, also when the cultural assets are not completely comparable. If the data of a new asset are added to the model, it will be verified whether the individualized risk prediction for this asset (high risk, low risk, unclear) is aligned with the individualized risk prediction for the existing assets (based on a selection of a subset of the overall data, and—where applicable—results reported in the literature). If an asset does not fit into the scheme than its profile is analyzed for key data that could cause these differences in the light of the already processed asset profiles. This leads to a constant re-analysis of the entire sample of the asset profiles with the goal to increase homogeneity and to identify irrelevant variables but without eliminating them as they may become relevant in other configurations that have not yet been identified.

The software Race, that implements the introduced mathematical model, has a general architecture that allowed applications in every context where causal detection and risk analysis is needed [7,10,11,12,13,14]. Table 1 shows these applications and organizations that provided application data and made associated experiments and applicative analysis.

## 6. Experiments

**Figure 13:**
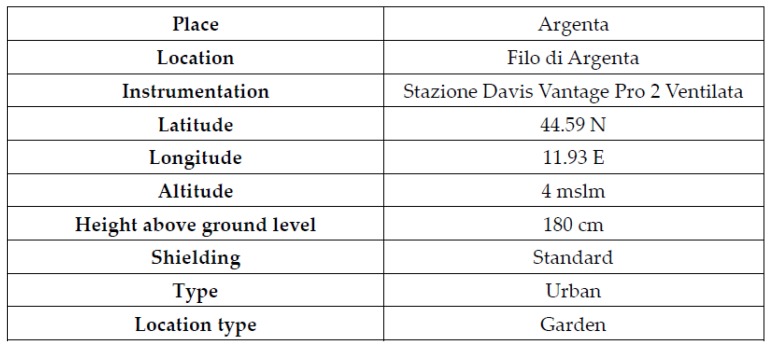
Descriptive information of the meteorological station of Filo d’Argenta. Reprinted from http://my.meteonetwork.it/station/ero299/stazione.

Low-cost gas sensors get more and more interest in the field of air pollution and one of the hottest topics is the calibration of them starting from data coming from more complex and expensive devices [34,35,36,37,38]. We planned an experiment to prove the generality of our causal engine. The experiment was carried using data (250 values) coming from the sensor located in Bando d’Argenta (44°38′56.40″ N, 11°53′24.98″ E) [39]. The considered gas variable is NO_2_ and data relative to the analyzer of the local station and the low-cost sensor are considered. We considered also meteorological variables coming from the meteorological station of Filo d’Argenta, near to Bando d’Argenta (see Figure 13). The measuring campaign was performed from March 2016 to December 2018.

**Figure 14:**
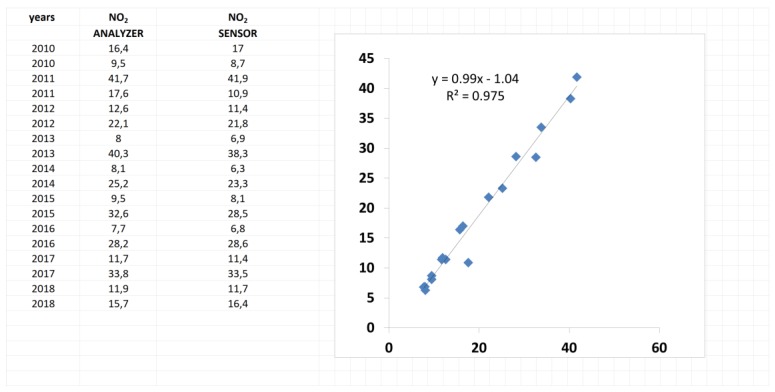
Linear relationships between NO_2_ measurements coming from the mean value of the considered low-cost sensor and the local analyzer.

First step: we considered linear regression between the two series of data (low-cost sensor data and analyzer data) and the output was a strong linear relationship between these two series (see Figure 14). We considered, for the value of the sensor, an average value for a period of five days and the central value for the analyzer.

To better understand the result, we submitted this distribution to our causal engine. The output is that to explain the distribution and then the punctual delay between the low-cost sensor and the analyzer we need to understand and to discover a hidden variable that is a composition of two or more elementary variables, not uncorrelated in their effects. The hidden variable is the output of the flex described in Figure 2. This last concept will become clear in the rest of the experimental phase.

**Figure 15:**
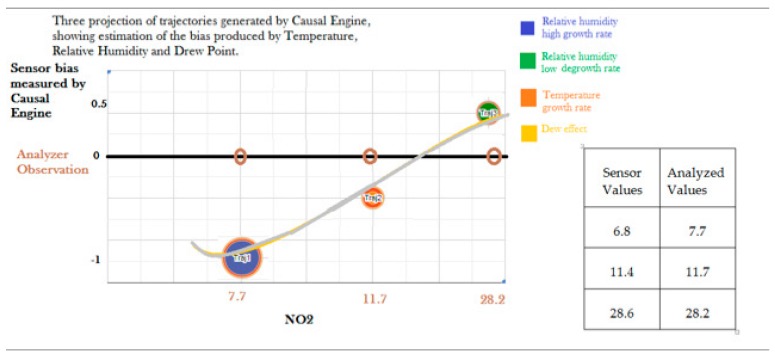
Three trajectories produced by the causal engine. The table represents punctual values of observed NO_2_ by the low-cost sensor and the analyzer. The effective value of the low-cost sensor is inferred considering together all three variables (temperature, relative humidity and dew). This could explain the reason behind the apparently random behavior of errors in many datasets.

We reviewed literature analyzing the problem of calibration of low-cost sensors measuring air pollution. In particular, we focused in the paper [40]. In this study, to improve the calibration of low-cost sensors, the focus is on meteorological variables and, in particular, two variables are considered to reduce the calibration error: temperature and humidity. The causal engine results tell us that, if a hidden variable is a composition of two elementary variables, an inverse relationship with the target gas variable (NO_2_) is required for these two variables. In this case, [40] reports a negative correlation between temperature and all considered gas variables and a positive correlation between relative humidity and all gas variables. This is a first confirmation of the output of the causal engine. We collected all meteorological data coming from the considered meteorological station and we obtain a new data distribution (that includes temperature and relative humidity) that we submit to the causal engine. In the previous regression, a punctual maximum error of 0.9 for the NO_2_ value is reduced to 0.5. The averaged error is 0.27.The analysis of produced trajectories in the output of the causal engine defines a non-linear relationship between the divergence between low-cost sensor values and analyzer values (Figure 15). In particular, we note two main relationships: first, a rapid grown in the relative humidity has a negative effect in the sensor value and then a lower value compared with the true value (R^2^ = 0.83 considering rapid grown in relative humidity, i.e., more than 15% and sensor error, i.e., the absolute difference between the analyzer value and the sensor value); second, the same effect can be observed also with a grown in the temperature but to understand the precise effect of temperature the dew point variable has to be considered. In particular, a low relative change of the dew point amplifies the effect of small temperature changes. To show how the causal engine works, below is an example with three trajectories.

**Figure 16:**
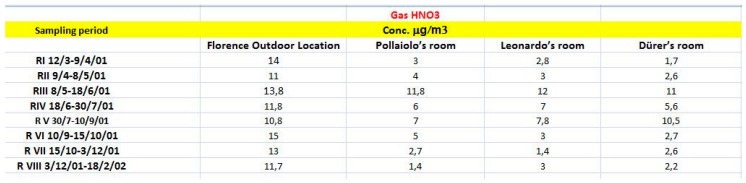
Data coming from three rooms of the Uffizi Gallery in Florence, in eight observation periods.

The analysis of relationships between low-cost sensor errors and external air composition is the worst case in terms of the complexity of causal relationships. In an open environment, many factors could cooperate to introduce errors in the sensor measurements and it could be difficult to understand them. The presence or absence of a monument or cultural asset does not modify this scenario. However, we proceeded with a new experiment closer to the field of cultural heritage, in a delimited and closed environment. Data come from the experiment documented in [41]. The physical location is the Uffizi Gallery in the Uffizi Palace of Florence, in Italy. The Gallery has a central air ventilation and conditioning system and three rooms (no. 9, 15 and 20) located in the same wing of the gallery, with different sizes (the order is 15 > 9 > 20). The sampling campaign was carried out by monitoring ozone, sulfur dioxide, nitrogen dioxide, nitrogen oxides, nitrous and nitric acids for eight periods from March 2001 to February 2002. Figure 16 reports the dates for each exposure period, and the value of nitric acids used for causal analysis in the outdoor location and in the three rooms.

The analysis of produced trajectories in the output of the causal engine to predict the risk of the pollutant agent in the three rooms, defines three hidden causal evidences: the level of exposure to outdoor air, that is high for Leonardo’s room and for Pollaiolo’s room but low for the third room; the exposure to the air conditioning system that affects the Pollaiolo’s room fist of all and in the lower level the Leonardo’s room and the Durer’s room; the exposure to visitors that affects Durer’s room and to a lesser extent the others two rooms. We use an estimation of the three variables for the three rooms to standardize measured data and we made a linear regression on standardized data obtaining a good level of precision (R^2^ = 0.78). The conclusion is that these three variables are to be collected to better understand the exact composition of the air in the three analyzed rooms.

## 7. Conclusions

Conservation and restoration of cultural heritage is something more than a simple process of maintaining the existing. It is an integral part of enhancing the cultural asset and cannot be viewed without considering the specific area where it is located. The social context that makes the ecosystem of the cultural asset has to play a role. Starting from this ambitious concept we tried to develop a model for the protection and enhancement of an area where a cultural heritage is hosted.

Three groups of properties characterize the model and its scientific contribution:

1. we proposed a new family of smart portable sensors tattoo, designed for analysis of cultural heritage surfaces. These tattoo prototypes present two innovation aspects, as: the miniaturizable tools, due to the high technological printers, and highly sensitive nanostructured layers, for a selective molecular recognition pathways on the tattoo sensors transducer.
2. we proposed the capability of the monitor system to become the new skin of the cultural assets and then their interface with the environment; the full integration with the ecosystem that the cultural heritage defines in an area. The monitoring system enters in the ecosystem proving it with new opportunities. This is possible thanks to a causal engine able to integrate heterogeneous data (physical and social data); examples of the ecosystem and social data are the physical infrastructure where the asset is located, its visitors, their behaviors, the presence of unattended events;
3. we proposed a complex and complete model for monitoring a cultural asset, considering all aspects behind a distributed monitoring system: a distributed network of sensors, communication channels, data and data analysis tools. We proposed a model, which allows keeping all together.

A first experiment, developed with a limited set of data available, shows the potential of the causal engine to understand complex causality relationships and then to allow the full use of available devices. In this experiment, we compared data coming from a low-cost sensor and the same data captured by a precise device. We show that, using meteorological variables, we can explain differences between the two sources with a new better level of precision. In particular, using the causal engine, we discover the significance of the dew variable. A second experiment regarding more explicitly cultural heritage has been done to understand the rules that allow understanding air composition in the rooms of a gallery. Two more steps need to be followed to enforce showed results. The first is an experiment that keeps together few precise and expensive sensors and many distributed low-cost sensors to show the capabilities of the causal engine to manage complex sets of data and to learn to start from them. In this scenario, the calibration of low-cost sensors has to consider also social variables, which can act as causal evidence of anomalous behavior. A mathematical model has to output the location of the precise sensors, in order to locate homogeneous areas in terms of calibration parameters for low-cost sensors.

The second is the repetition of the experiment with a large amount of available data and with more variables to refine the analysis of residuals. In particular new variables have to be searched not only in the set of chemical variables but also in the set of social and local variables. These last categories can be used to explain complex chemical behaviors. This allows us to reinterpret apparently random behaviors of errors in many existing datasets regarding air pollution and critical for decision-makers [42].

## Figures and Tables

**Table 1:** shows pasted applications of the race software for causal analysis and risk prevention.

Experiment or Application	Involved Organizations
Cardiovascular disease (CVD) risk prevention—first experimentation [10,12].	Dep. Medical Sc.—UCO Cardiology Inst.—Policlinico Tor Vergata, Rome, Italy, IRCCS San Raffaele Pisana, Rome, Italy, Clinical Institute Humanitas, Milan, Italy.
CVD risk prevention (integration with epigenetic data)—second experimentation [12].	Policlinico Tor Vergata, Rome, Italy
Diabetic Foot Ulcers risk prevention.	Policlinico Casilino, Rome, Italy.
Picaso Project (www.picaso-project.eu) [7].	Diagnostic imaging dep.—Policlinico Tor Vergata—Rome, Italy, Policlinic of Rheumatology and Hiller Research Unit—Heinrich-Heine University Dusseldorf—Germany
CVD–AMI (Acute myocardial infarction), genetic polymorphisms selection [13].	Dep. Of Health Sciences—A.A.Univ. Piemonte Orientale—Italy, Osp. Carità Maggiore, Novara—Italy—Ospedali riuniti Verbano Cusio Ossola—Italy
Pharmacological trials using nanocarrier sensors [11].	Dep.of Biotechnology, Chemistry and Pharmacy—University of Siena—Italy. Dep. Chemistry and Drug Technology—University La Sapienza—Rome—Italy
